# A Meta-Analysis of the Effect of Subthalamic Nucleus-Deep Brain Stimulation in Parkinson's Disease-Related Pain

**DOI:** 10.3389/fnhum.2021.688818

**Published:** 2021-07-01

**Authors:** Yu Diao, Yutong Bai, Tianqi Hu, Zixiao Yin, Huangguang Liu, Fangang Meng, Anchao Yang, Jianguo Zhang

**Affiliations:** ^1^Department of Neurosurgery, Beijing Tiantan Hospital, Capital Medical University, Beijing, China; ^2^Beijing Key Laboratory of Neurostimulation, Beijing, China

**Keywords:** Parkinson diseases, pain, follow-up, deep brain stimulation, meta-analysis

## Abstract

Pain from Parkinson's disease (PD) is a non-motor symptom affecting the quality of life and has prevalence of 20–80%. However, it is unclear whether subthalamic nucleus deep brain stimulation (STN–DBS), a well-established treatment for PD, is effective forPD-related pain. Thus, the objective of this meta-analysis was to investigate the efficacy of STN-DBS on PD-related pain and explore how its duration affects the efficacy of STN-DBS. A systematic search was performed using PubMed, Embase, and the Cochrane Library. Nine studies included numerical rating scale (NRS), visual analog scale (VAS), or non-motor symptom scale (NMSS) scores at baseline and at the last follow-up visit and therefore met the inclusion criteria of the authors. These studies exhibited moderate- to high-quality evidence. Two reviewers conducted assessments for study eligibility, risk of bias, data extraction, and quality of evidence rating. Random effect meta-analysis revealed a significant change in PD-related pain as assessed by NMSS, NRS, and VAS (*P* <0.01). Analysis of the short and long follow-up subgroups indicated delayed improvement in PD-related pain. These findings (a) show the efficacy of STN-DBS on PD-related pain and provide higher-level evidence, and (b) implicate delayed improvement in PD-related pain, which may help programming doctors with supplement selecting target and programming.

**Systematic Review Registration:** This study is registered in Open Science Framework (DOI: 10.17605/OSF.IO/DNM6K).

## Introduction

Pain is a common non-motor symptom affecting 20 to 80% of patients with Parkinson's disease (PD) (Koutoukidis et al., 2021). On the basis of disease onset, pain from PD can be divided into three types: (i) PD directly related pain: pain is related to the onset, symptoms, or treatment of PD; (ii) PD indirectly related pain: patients suffer from chronic pain before the onset of PD, and the symptoms or treatment of PD aggravate the original pain; and (iii) PD unrelated pain: pain is neither caused nor aggravated by PD. The first two categories are usually called PD-related pain (Mylius et al., 2015, 2021). PD-related pain may be categorized into several subtypes, such as musculoskeletal, dystonic, radicular neuropathic, and central pain, and can greatly reduce the quality of life of a patient. Certain types of pain from PD, such as musculoskeletal and dystonic pain, may respond to manipulation of dopaminergic medication (Drake et al., 2005; Ha and Jankovic, 2012), yet on–off phenomena and side effects, such as dyskinesia, still mean that patients suffer from pain (Stefani et al., 2013; Karnik et al., 2020). Subthalamic nucleus (STN) deep brain stimulation (DBS) is a well-established treatment for PD and is suggested to alleviate pain in patients with PD (Drake et al., 2005; Ha and Jankovic, 2012; Sugiyama et al., 2015).

Some studies have reported the efficacy of STN-DBS on PD-related pain, and it is believed that STN-DBS can improve the sensory and pain thresholds of a patient (Marques et al., 2013; Tseng and Lin, 2017). However, current DBS clinical studies on PD-related pain still have shortcomings, with most including a retrospective design, limited sample size, and single-arm studies. There is currently no high-level evidence confirming the efficacy of STN-DBS on PD-related pain. Some patients also show no relief in pain after DBS treatment (Kim et al., 2011; Karnik et al., 2020). Therefore, the curative effect of STN-DBS on PD-related pain is not unequivocal. At present, there is only one study reporting the long-term effect (8 years) of STN-DBS on PD-related pain (Jung et al., 2015), yet if STN-DBS can improve pain, will its curative effect weaken over time? Exploring this question may greatly facilitate the identification of patients who could benefit from DBS and help enhance the understanding of how STN-DBS affects PD-related pain. Thus, in this study, we aimed to explore the efficacy of STN-DBS on PD-related pain and the effect of short-term and longer follow-ups.

## Method

### Search Strategy

**Figure 1:**
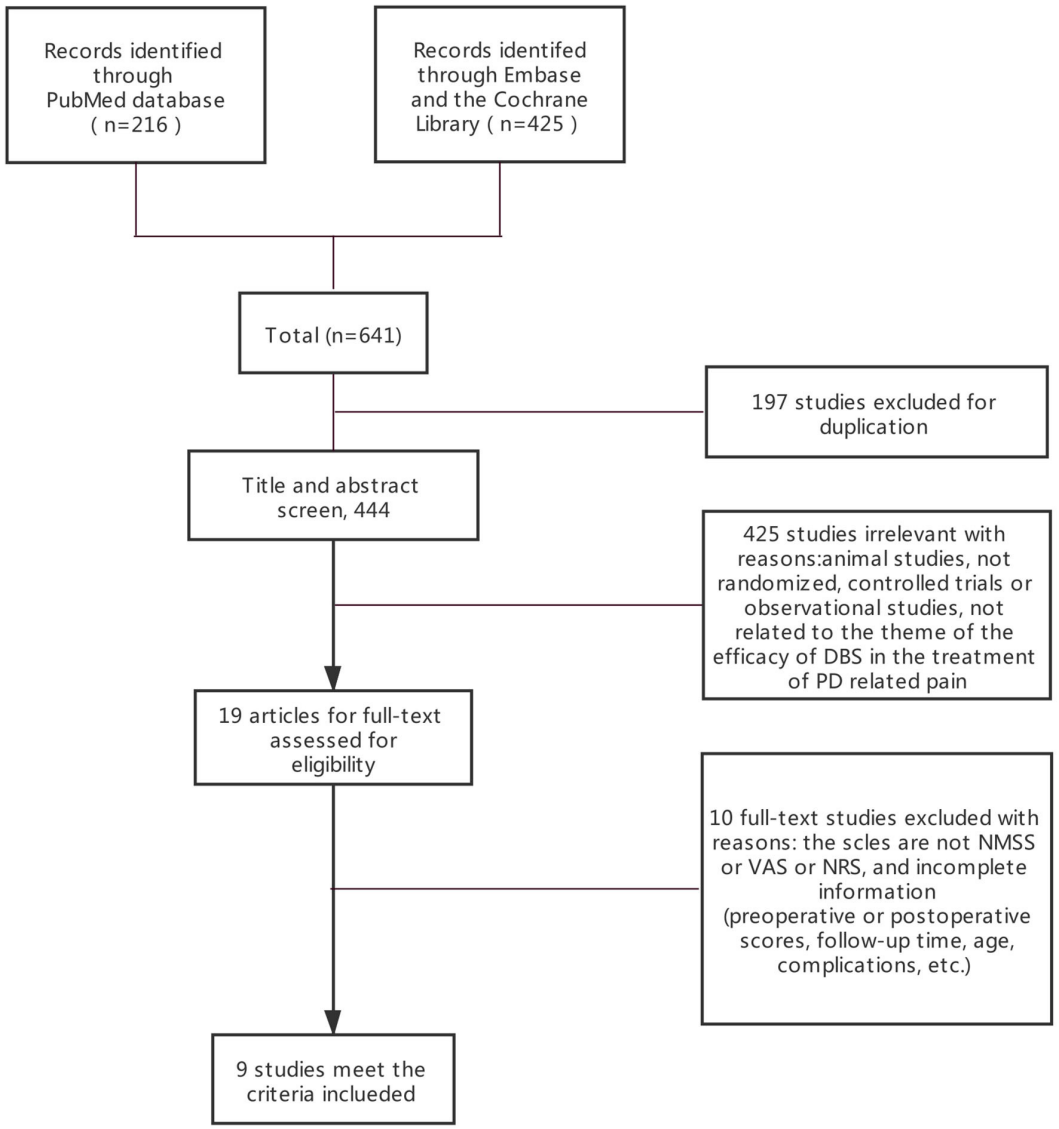
PRISMA flowchart: A flowchart ultimately showing the included studies. PRISMA, preferred reporting items for systematic reviews, and meta-analyses.

Three electronic databases (PubMed, Embase, and the Cochrane Library) were searched following the Preferred Reporting Items for Systematic Reviews and Meta-Analyses (PRISMA) guideline. The final search was performed in September 2020. We searched all articles related to DBS for pain from PD. The following search terms were used: [“Parkinson disease (MeSH),” “Parkinson” or “Parkinson's disease”] and [“pain (MeSH),” “deep brain stimulation (MeSH),” or “DBS” or “STN-DBS”]. We did not limit age, sex, or operative time. A flow chart of the literature search is shown in Figure 1. This study is registered in Open Science Framework (DOI: 10.17605/OSF.IO/DNM6K).

### Inclusion and Exclusion Criteria

The inclusion criteria for eligible studies were as follows: (1) subjects were patients with PD who were treated with STN-DBS and regularly taking drugs for PD. (2) The studies were published in English. (3) PD-related pain was classified using the Ford 2012 classification (Sophie and Ford, 2012). (4) The studies reported any objective NRS (10 points, 0 = no pain, 10 = worst pain), VAS (10 cm, 0 mm = no pain, 100 mm = worst pain, or 0 cm = no pain, 10 cm = worst pain), or NMSS scores at baseline and at the last follow-up visit to determine the efficacy of STN-DBS on PD-related pain. (5) The studies described the characteristics of pain related to PD, such as the number of patients with PD-related pain. Regarding the efficacy of DBS, the studies had to meet criteria (1), (2), (3), and (4). If the studies meet criteria (5), the efficacy for this cohort would be included. The studies focused on the effects.

### Data Extraction

A data extraction template was used to build an evidence table that included the following items: author, publication year, number of patients, duration of disease in years, and follow-up time, Unified Parkinson's Disease Rating scale part-III (UPDRS-III), Hoehn and Yahr (H&Y), levodopa equivalent daily dosage (LEDD), and VAS/NRS or NMSS at baseline and last follow-up visit. All VAS/NRS or NMSS scores were assessed in the condition in which the patients were under their regular medication with the stimulator turned “on”. Two authors (Yu Diao and Yutong Bai) independently extracted the data.

### Statistical Analysis

First, all scores of NRS, VAS, and the miscellaneous domains of NMSS were only compared with pre-operation and follow-up scores. An unstandardized b coefficient with 95% confidence interval (CI) between changes in VAS/NRS and changes in NMSS was estimated for each study assuming a linear relationship between them. For VAS studies, the reporting of pain scores was different than that of NRS scores; therefore, all outcomes were converted into NRS scores as normalization to better demonstrate pain (Chen et al., 2020). Second, outcomes from short-term follow-up (<6 months) and longer follow-up (≥6 months) were compared. VAS/NRS scores were generated using the standardized mean difference (SMD) with 95% CI, while the miscellaneous domains of NMSS used the mean difference (MD) with 95% CIs. Negative values for pain severity represent pain reduction, while positive values represent pain augmentation. Random effects models were used. Heterogeneity between studies was quantified using the index of heterogeneity (*I*^2^), and the effect size was measured using the model of Hedge. In studies with more than one follow-up point, the outcomes of the longest follow-up point were chosen. All analyses used STATA 16.0, and the significance threshold was set at *P* <0.05. Finally, according to the Cochrane Handbook for Systematic Reviews of Interventions 4.2.5 (http://www.cochrane.org/resources/handbook/hbook.htm), because only nine studies were included, publication bias was not done.

### Quality Evaluation

The Newcastle–Ottawa Scale (NOS) was used to assess the quality of the studies and included the following evaluation criteria: adequacy of the case definition, representativeness of the cases, selection of controls, definition of controls, comparability of cases/controls, the same method of ascertainment, and non-response rate. For quality assessment, NOS total score ranged from 0 star (lowest quality) to 9 stars (highest quality). Usually, a study with seven or more stars was classified as a high-quality study (Koutoukidis et al., 2021). All the articles chosen were of middle to high quality.

## Results

### Search Results

The systematic search returned 641 entries for screening. Nine studies involving 338 patients met the criteria and were included by reviewing the full text of the articles (PRISMA diagram in Figure 1). Among the included patients, 12 received unilateral STN-DBS treatment. Since the number of patients who received unilateral STN-DBS was small and the authors did not distinguish between unilateral and bilateral STN-DBS, we did not compare the effects of unilateral and bilateral differences. After excluding articles that did not conform to the eligibility criteria, two randomized controlled trials (RCTs) and seven observational studies were included (Figure 1). Risk of bias (quality) assessment and detailed information of all the nine studies are shown in Tables 1, 2.

**Table 1:** Summary of critical appraisal of included studies using the Newcastle-Ottawa Scale for assessing the quality of the studies included.

	**Selection**	**Comparability**	**Outcome**	
Kim et al. (2012) Korea	※※		※	Mid
Kim et al. (2008) Korea	※※		※	Mid
Hwynn et al. (2011) USA	※※		※	Mid
Cury et al. (2014) Brazil	※※※		※	Mid
Jung et al. (2015) Korea	※※※		※	Mid
Fabbri et al. (2017) Portugal	※	※	※	Mid
Dafsari et al. (2020) France	※※	※※	※	High
Gong et al. (2020) China	※※	※	※	Mid
Jost et al. (2020) Germany	※※	※※	※	High

*Each of these three categories has further subcategories and gives ※. The studies with the maximum number of ※ are of higher quality than those with fewer ※. Empty cells show that no ※ is available for this category*.

**Table 2:** Details of studies included in the meta-analysis of DBS in the treatment of PD-related pain.

**Study, year country**	**Total N**	**Follow-up (months)**	**Duration (years)**	**UPDRS_III (pre-med off)**	**UPDRS_III (pre-med on)**	**H and Y**	**LEDD**	**Operation**	**Outcome measures**
Kim et al. (2012) Korea	21	24 (short-term follow-up 3m)	10.6 ± 4	32.4 ± 9.8	18.5 ± 10.3	2.9 ± 0.9	752.5 ± 400.3	STN-DBS	NRS, parts of the body, quality of pain
Kim et al. (2008) Korea	29	3	9.9 ± 4.6	34.1 ± 12.2	20.6 ± 13.5	2.9 ± 0.9	736.2 ± 374.3	STN-DBS	NRS, parts of the body, quality of pain
Hwynn et al. (2011) USA	10	12.1 ± 7.3	9.9 ± 2	_	_	_	_	unilateral STN-DBS, unilateral GPi-DBS	NMSS
Cury et al. (2014) Brazil	44	12	15 ± 7.6	41.5 ± 11.2	16.0 ± 9.0	2.8 ± 0.64	1092 ± 456	STN-DBS	NMSS, VAS, parts of the body, quality of pain
Jung et al. (2015) Korea	24	96(short-term follow-up 24 m)	18 ± 3.8	35.9 ± 14.7	19.7 ± 13.6	2.8 ± 0.7	850.3 ± 449.2	STN-DBS	NRS, parts of the body, quality of pain
Fabbri et al. (2017) Portugal	32	55.2 ± 15.6	18.7 ± 5.1	_	_	_	1178.8 ± 553.9	STN-DBS	VAS, NMSS
Dafsari et al. (2020) France	75	_	11.3 ± 5	42.2 ± 9.6	_	_	1195.6 ± 459.4	STN-DBS and GPi-DBS	NMSS
Gong et al. (2020) China	36	3 or 6	<8–10	29.0 ± 10	_	_	_	STN-DBS and GPi-DBS	NRS and quality of pain
Jost et al. (2020) Germany	67	36	10.8 ± 4.9	_	_	_	1199.1 ± 587.5	STN-DBS	NMSS

*UPDRS, The Unified Parkinson's Disease Rating Scale; LEED, levodopa-equivalent daily dose; NRS, numerical rating scale; VAS, visual analog scale; NMSS, non-motor symptom scale*.

### Quality Evaluation and Baseline Characteristics

Two of the RCT studies were of high quality, while the others are single-arm ones and lack a control group with less scores. All articles evaluated by NOS were of middle to high quality (Table 1). Detailed information of patients at baseline is provided in Table 2.

### The Effectiveness of STN–DBS on PD-Related Pain

**Figure 2:**
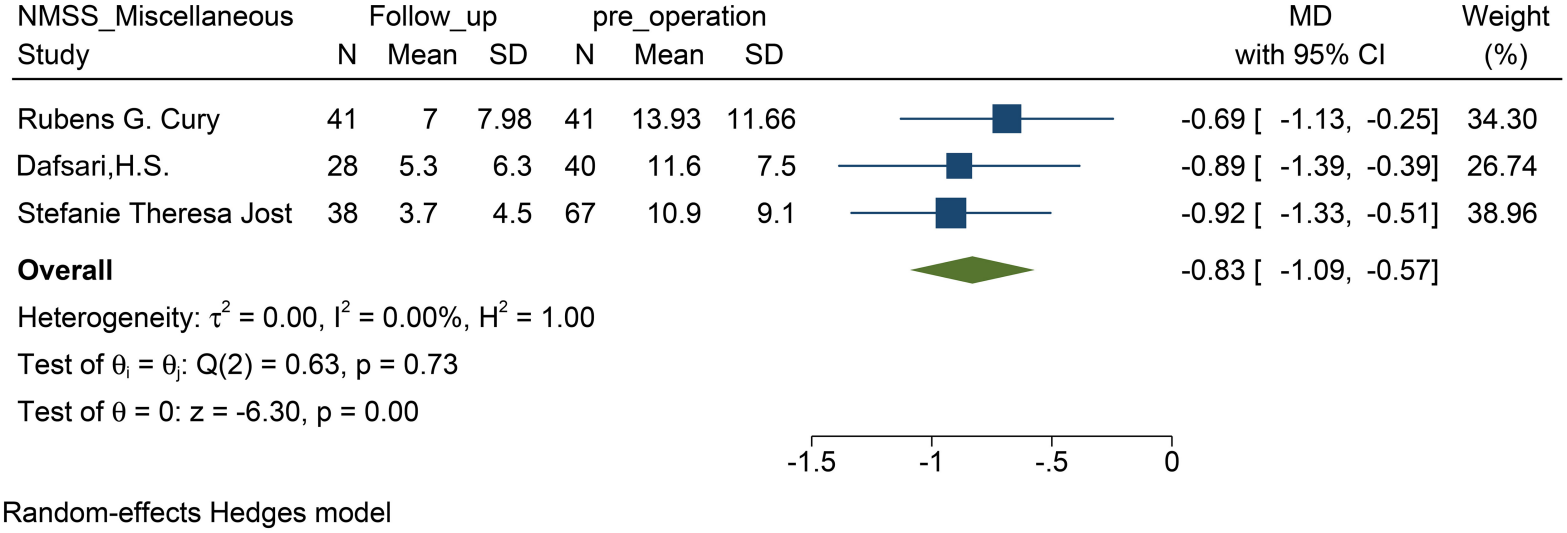
Forest plots of pain severity: pain severity assessed by the score of miscellaneous domains including pain in the NMSS, showed a significant reduction after STN-DBS. NMSS, non-motor symptom scale; STN-DBS, subthalamic nucleus deep brain stimulation.

**Figure 3:**
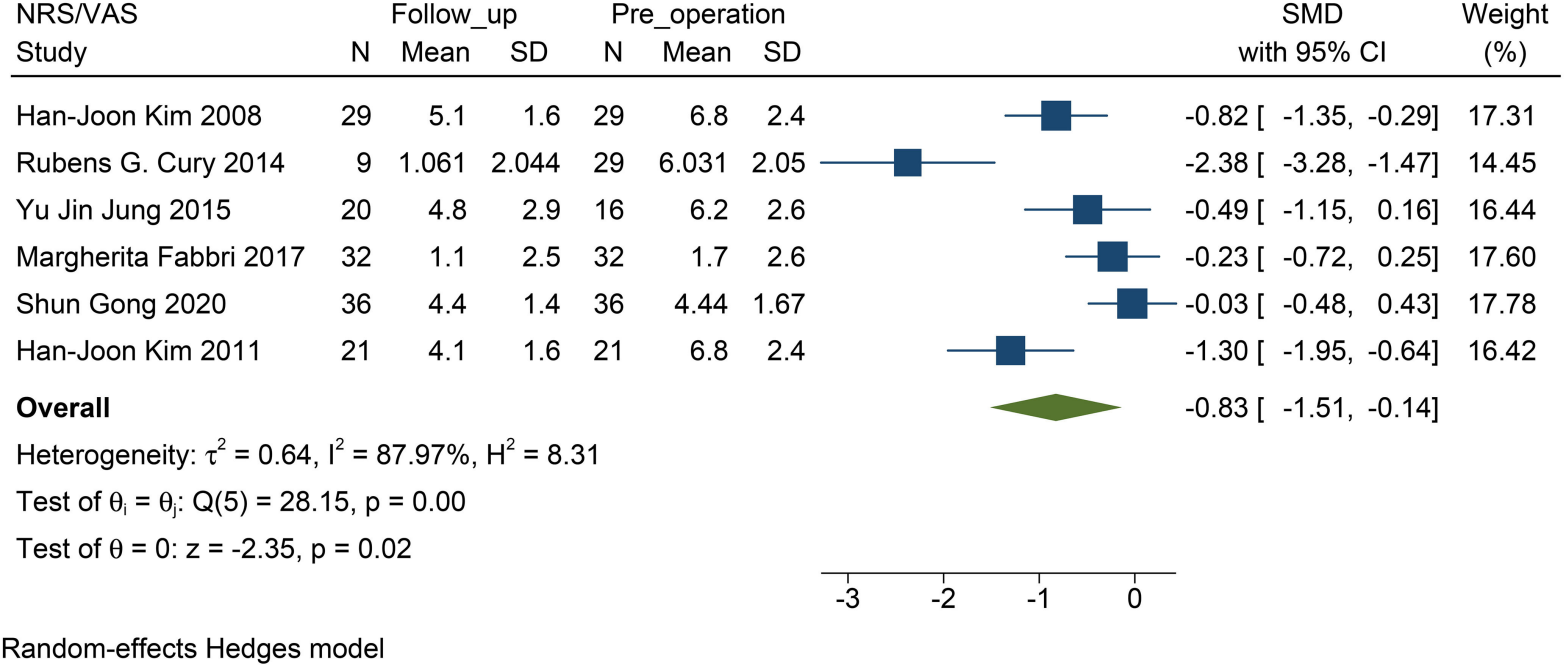
Forest plots of pain severity: pain severity assessed by the VAS/NRS decreased significantly after STN-DBS. NRS, numerical rating scale; VAS, visual analog scale; STN-DBS, subthalamic nucleus deep brain stimulation.

STN-DBS, both NRS and VAS scores were significantly decreased in the patients. Similarly, the miscellaneous domain score (including pain) of the NMSS was significantly decreased. The MD of the miscellaneous domains changed by 0.83 on average (95% CI: −1.09 to −0.57, *p* <0.01, *I*^2^ = 0 %, *n* = 3) (Figure 2); while standardized mean difference (SMD) of the NRS and VAS scores changed by 0.83 on average, and this item was found to have high heterogeneity (95% CI: −1.51 to −0.14, *p* = 0.02, *I*^2^ = 87.97%, *n* = 6) (Figure 3). However, because of the limited number of studies, publication bias in the NRS, VAS, and NMSS results is objective.

Besides, we also analyzed four of the studies on types of PD-related pain. It was found that DBS had an effect on musculoskeletal and dystonic subtypes of pain (Cury et al., 2014). However, due to differences in statistical methods of pain subtypes in different studies, one counted the changes in the number of pain patients before and after DBS, while one did not record the pain classification of postoperative patients. Although the other two have counted the number of painful parts, the distinction was different. For example, one counted the head and neck as one part, while another discussed the head and neck separately, so we only listed each study in Table 3.

**Table 3:** The number of pain (pre) means before surgery, and (DBS) means after STN-DBS.

	**Neuropathic-radicular** **(pre)**	**Neuropathic-radicular** **(DBS)**	**Musculoskeletal** **(pre)**	**Musculoskeletal (DBS)**	**Dystonic** ** (pre)**	**Dystonic** ** (DBS)**	**Central** ** (pre)**	**Central** ** (DBS)**	**Classification method**
2008 Korea	8	5	25	16	9	9	25	23	Number of pain location
2014 Brazil	2	2	26	5	14	1	2	1	Number of pain patients
2015 Korea	1	1	5	3	3	0	5	3	Number of pain location
2020 China	1	–	29	–	9	–	4	–	Number of pain patients

### Comparison Between <6 Months and ≥6 Months Outcomes

**Figure 4:**
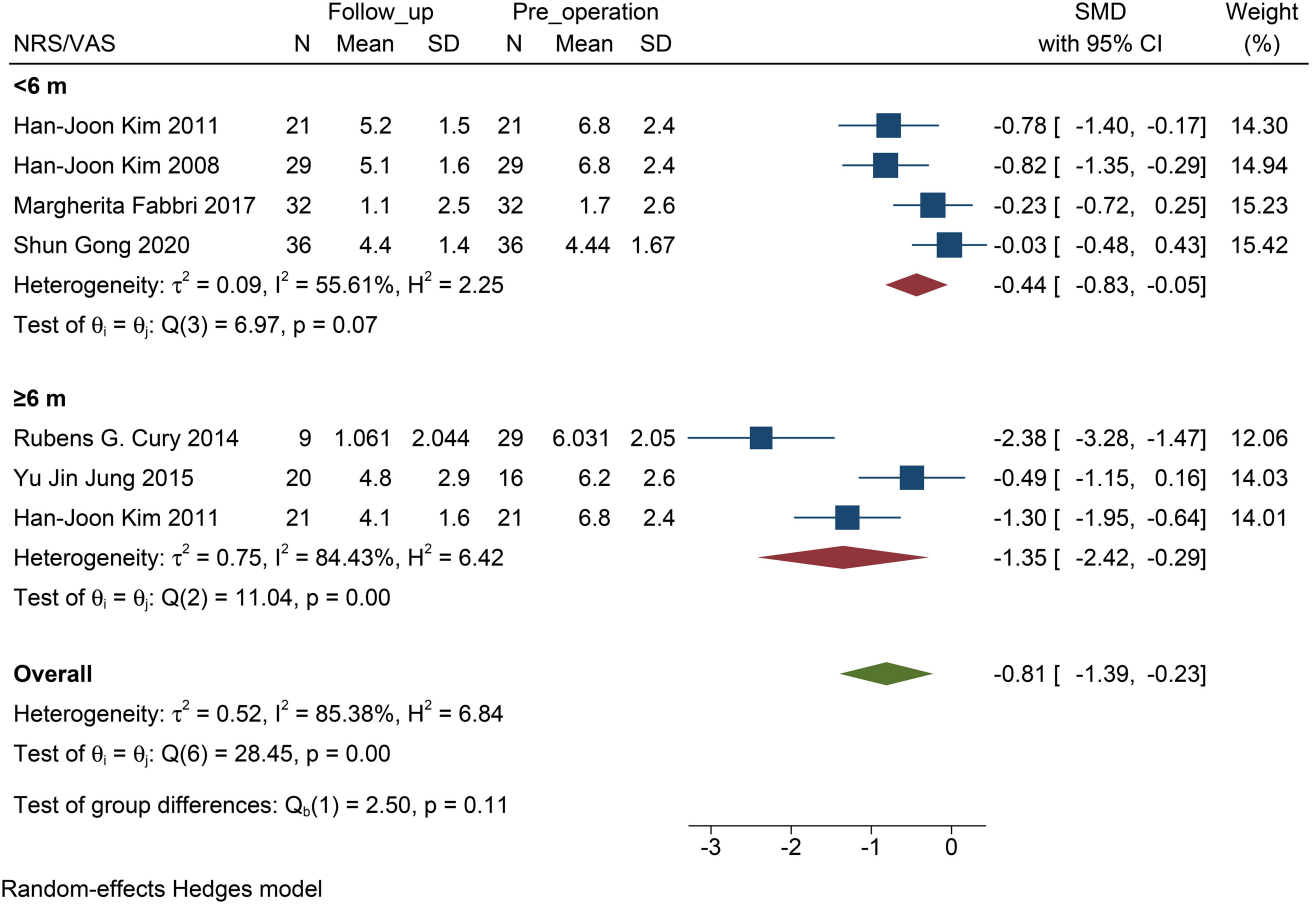
Forest plots of pain severity with PD follow-up subgroups: The SMD of pain severity divided into two subgroups by the time of follow-up accessed by the score of NRS/VAS. Only the long-term subgroup has statistical significance. PD, Parkinson's disease; NRS, numerical rating scale; VAS, visual analog scale; SMD, standardized mean difference; N, simple size.

The NMSS score was not included in this analysis, because the size of the studies was small, and the follow-up time of the patients was ≥6 months. For NRS/VAS, the decrease in pain was statistically significant only when the follow-up exceeded 6 months (*P* <0.01, Figure 4). There was no statistical significance when the follow-up time was <6 months (*P* = 0.07, Figure 4). No statistical difference was found between the two subgroups (*P* = 0.11).

**Figure 5:**
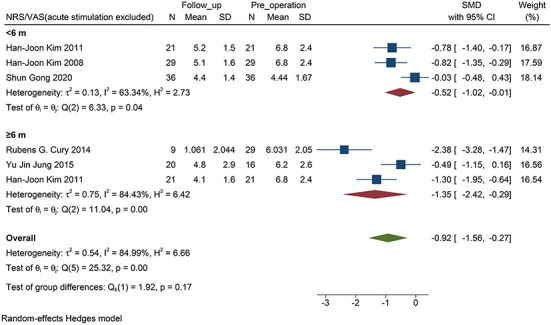
Forest plots of pain severity with PD follow-up subgroups: The SMD of pain severity accessed by the score of NRS/VAS excluded the accurate stimulation study, shows a significant change between baseline and follow-up visit in all subgroups. PD, Parkinson's disease; NRS, numerical rating scale; VAS, visual analog scale; SMD, standardized mean difference; N, sample size.

However, by excluding a study of acute stimulation (VAS assessment only in the two states when the stimulator was turned “on” and “off” immediately), the follow-up was statistically significant in the subgroup within 6 months (*P* = 0.04, Figure 5).

### Prevalence of Pain From PD

**Figure 6:**
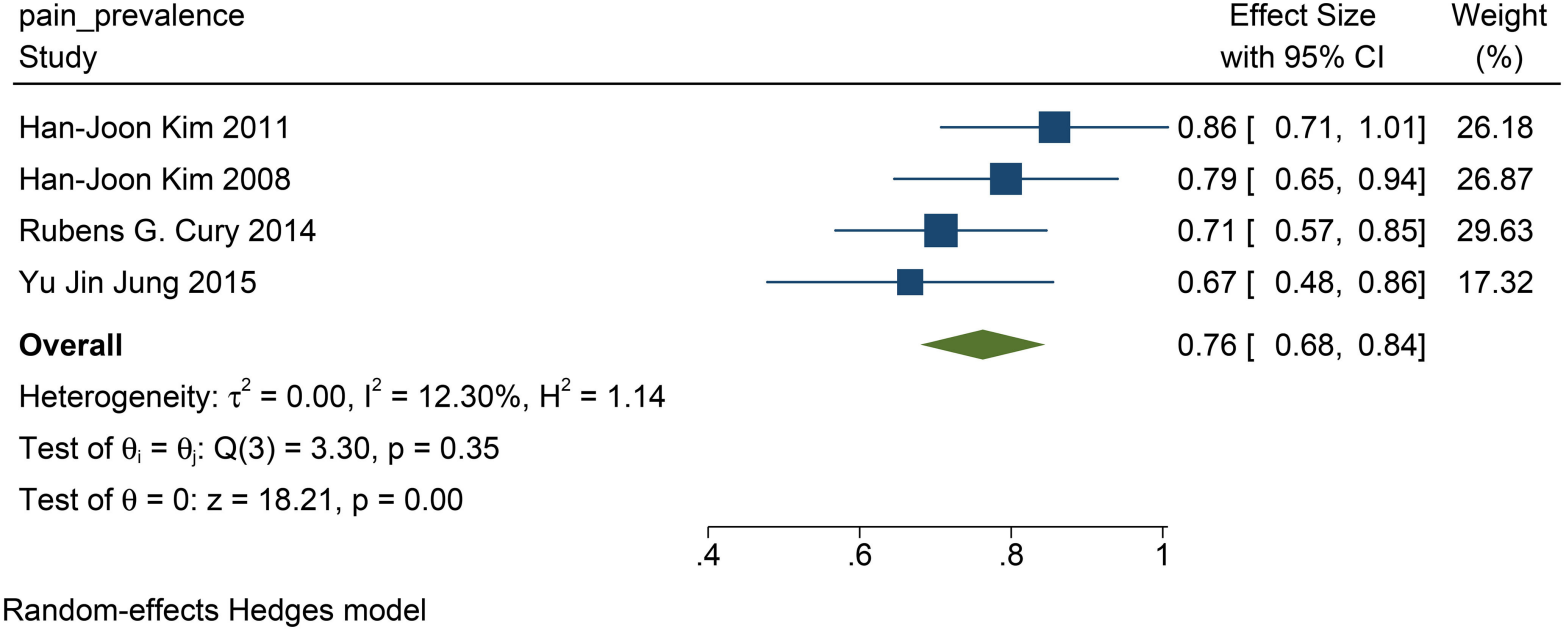
The pain prevalence in patients with PD at baseline: the pain prevalence in patients with PD at baseline is 76%. PD, Parkinson's disease.

**Figure 7:**
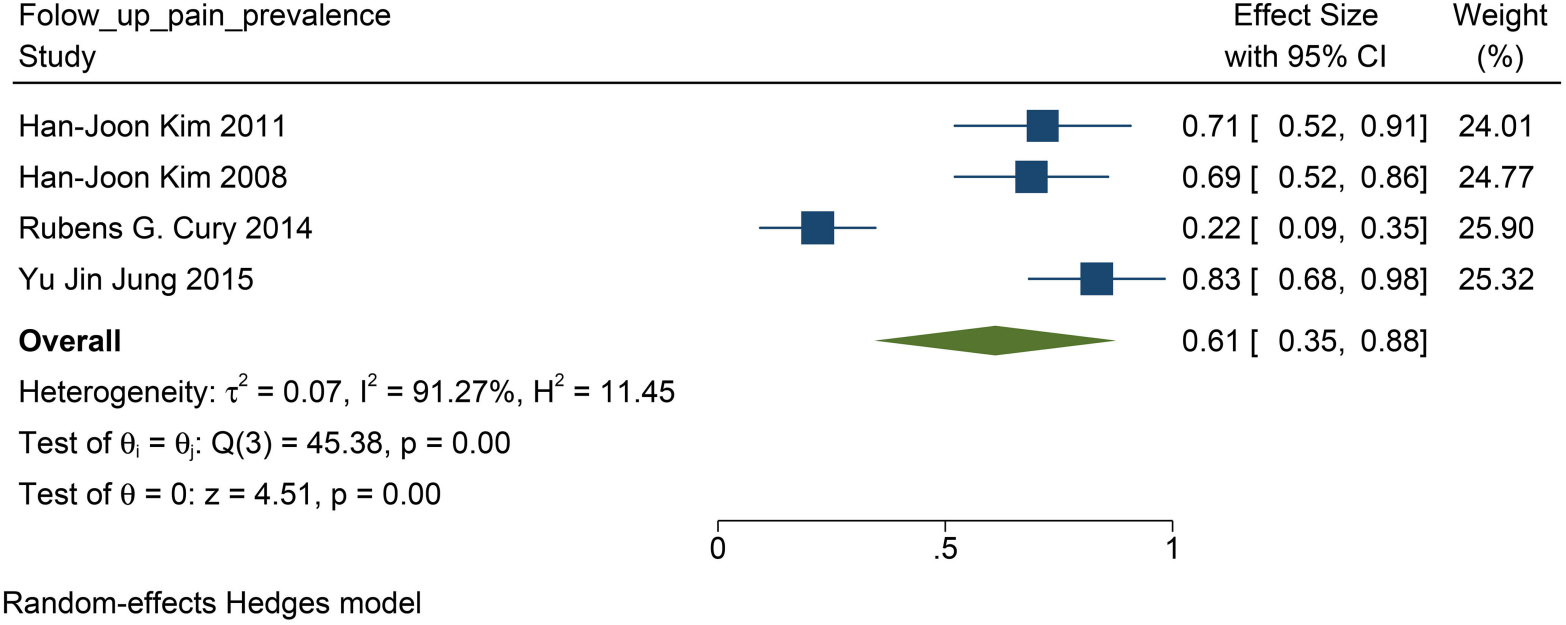
The pain prevalence in patients with PD at follow-up visit: the pain prevalence in patients with PD at the follow-up is 61%. PD, Parkinson's disease.

We found that in all the four studies researched, 76% of the patients before DBS had PD-related pain (95% CI: 0.68 to 0.84, *p* <0.01, *I*^2^ = 12.3 %, *n* = 4) (Figure 6), while 61% of the patients suffered from PD-related pain after STN-DBS (95% CI: 0.35 to 0.88, *p* <0.01, *I*^2^ = 91.27%, *n* = 4) after STN-DBS (Figure 7). In the postoperative pain study, one of the studies was followed up to 8 years, and the proportion increased compared with pre-operation. Thus, the results are heterogeneous.

## Discussion

This study has investigated the efficacy of STN-DBS on the treatment of PD-related pain. To the best of our knowledge, we have confirmed the efficacy of STN-DBS on PD-related pain and have provided a level of evidence corroborating its efficacy. This study is the first to find a delayed improvement in PD-related pain. Meanwhile, this study is pioneering in that we have found equivalence between a subjective scale (such as VAS/NRS) and the NMSS scale used for doctor consultation. The results show that STN-DBS can improve NMSS and VAS/NRS scores. The subgroup analysis showed that the pain improvement of patients in ≥6 months after surgery was clinically significant, whereas the pain improvement in ≤ 6 months was not statistically significant. After excluding the acute stimulation study, pain improvement within 6 months was also statistically significant. Therefore, when the programming doctor adjusts the parameters because of pain for the first time, the patient should be given sufficient advice to ensure they understand that the improvement in pain is delayed and that they need not be anxious, which may make the symptoms worse. Also, the doctor does not need to repeatedly adjust the parameters when the pain symptoms of the patient have not improved in time. In addition, in the long-term follow-up with the subgroups, we found that as the disease progressed, the pain prevalence increased but the pain scores remained below the baseline level, which suggests that STN-DBS can improve the pain symptoms of patients in the long run, but stimulation parameters should be improved to adapt to progression of the disease in the later stage or when new pain appears.

### The Efficacy of STN–DBS on PD-Related Pain

This study found that after STN-DBS, whether in the subjective evaluation scale (VAS or NRS) or in the scale evaluated by the doctor (NMSS), the pain of the patient was effectively relieved. Coincidentally, the change in both parameters reached 0.83 (Figures 2, 3), which indirectly proves that the subjective scale of NRS/VAS can be used to objectively evaluate the degree of pain in the patient (Karnik et al., 2020).

The pain and somatosensory thresholds of patients with PD are different from those of healthy controls (Sung et al., 2018). Moreover, STN-DBS can effectively change the pain threshold and somatosensory abnormalities of patients, thereby improving the pain symptoms of patients with PD (Cury et al., 2016; Kaszuba et al., 2019; Rukavina et al., 2019; Dogru Huzmeli et al., 2020). In addition, rat studies on STN-DBS in the treatment of PD-related pain also found that STN exhibited complex tonic and phasic responses to noxious stimuli (Pautrat et al., 2018). Some functional magnetic resonance imaging studies also show that the pain network (such as primary somatosensory and anterior cingulate cortex) is decreased by STN-DBS in patients whose pain was relieved by DBS compared with patients whose pain were not relieved by DBS (DiMarzio et al., 2019). In addition, the pathology of PD may influence pain in several ways. First, the basal ganglia receive nociceptive information from the thalamus and the amygdala. Neurons in the substantia nigra, caudate, putamen, and globus pallidus also respond to noxious stimulation. This might be why STN-DBS can reduce pain in patients with PD. Second, there might also be more direct effects on transmission of pain signals within the spinal cord *via* basal ganglia outputs to descending dopaminergic and serotonergic systems, while activation of γ-aminobutyric acid-mediated inhibition in the spinal cord might be influenced by STN-DBS so that pain is regulated (Conte et al., 2013). This may be the basis for STN-DBS to decrease the pain scores in NRS/VAS and NMSS of patients with PD. However, it should be emphasized that although we have cited two articles by the same author, the patients selected in the two articles were from two completely different groups of patients, so they can be analyzed independently (Kim et al., 2008, 2012).

In addition, this study placed unilateral and bilateral DBS together for analysis, because the studies we included did not discuss the different effects of unilateral and bilateral DBS on pain. To date, no studies have compared the differences between unilateral and bilateral STN-DBS in terms of clinical efficacy. In this study, we believe that both unilateral and bilateral DBS have curative effects on PD-related pain. Some studies have suggested that both sides can improve the motor symptoms of a patient and symptom fluctuations; therefore, DBS is effective for PD-related pain affected by motor symptoms (Conte et al., 2013; Gong et al., 2020). Some animal studies also suggest that regardless of whether it is bilateral or unilateral, STN-DBS increases mechanical thresholds and offers improvements to chronic pain in patients with PD (Sung et al., 2018; Kaszuba et al., 2019). Pain is a very complex symptom that incorporates changes in various aspects including an unpleasant sensory experience associated with actual physical damage, which is a crucial emotional and cognitive component. Therefore, improvement of pain is not achieved through one simple mechanism. Unilateral STN-DBS may improve the pain of a patient by improving motor symptoms, unilateral pain threshold, and so on, but whether it can improve pain in other pathophysiology or brain networks is still a question. Bilateral STN-DBS could improve the proficiency of inhibiting upper limb movements, and this change in inhibitory control plays a key role in shaping the individual response to pain (Frank et al., 2007; Mirabella et al., 2013). In addition, it has also been shown that unilateral STN DBS does not affect either the reactive (the ability to stop a response outright when a stop instruction is presented) or the proactive inhibition (the ability to flexibly adapt the motor strategy according to constraints embedded in the current context) of upper limb movements (Mancini et al., 2018). This may suggest that, compared with unilateral STN-DBS, bilateral STN-DBS has different or more mechanisms in controlling patient pain, especially in terms of crucial emotional and cognitive component. Bilateral STN-DBS may have a better curative effect, while unilateral STN-DBS is slightly weaker for the emotion or cognition that requires whole brain regulation.

### Short- and Long-Term Effects on PD-Related Pain

When STN-DBS stimulation was shorter than 6 months, we found that STN-DBS did not statistically reduce the NRS score (*P* = 0.07, Figure 4). Interestingly, in the four short-term efficacy studies included, one was a study on acute stimulation efficacy. When we excluded this study and evaluated the short-term effect of DBS within 6 months, a positive result was obtained (*P* = 0.02, Figure 5). Therefore, we suggest that the results are due to the following two aspects: (i) STN-DBS does not change the pain network in a short time. It requires long-term stimulation to reshape the pain network and gradually increase the pain threshold of a patient (Pautrat et al., 2018; Cury et al., 2020; Dogru Huzmeli et al., 2020). (ii) Usually, the network of pain in patients includes inner pathways related to emotions and outer pathways related to nociceptive stimuli. The poor effect of acute stimulation on pain may be an inability to immediately change the conduction of the two pathways (Greenspan et al., 1999; Blanchet and Brefel-Courbon, 2018).

In addition, long-term efficacy in patients with PD has high heterogeneity. When we excluded a 1 year follow-up study, the heterogeneity was markedly reduced. Therefore, we suggest that the efficacy of DBS can achieve a relatively stable efficacy in a year (Cury et al., 2014). Further, in the 2 and 8 years follow-up studies, the pain score was still decreased, which confirms that the effect of STN-DBS on PD-related pain persists for a longer follow-up (up to 8 years). The pathological brain network of patients with PD can be normalized after DBS stimulation, which is closer to that of healthy controls. We speculate that a network of pain may also represent such change (Horn et al., 2019).

PD is a progressive disease. The progression can lead to new pain or aggravation of the original pain (Jung et al., 2015; Kaszuba et al., 2019). However, we found that STN-DBS has a long-term and delayed effect on pain. On the basis of the result, we suggest that doctors can avoid repeated programming when there is no significant improvement in pain. Second, doctors can perform stimulation for an extended period and can comfort the patient while waiting for its effect to reduce their psychological expectations, stabilize their emotions, and avoid aggravation of their symptoms caused by emotional problems.

### The Impact of STN–DBS on Prevalence With PD-Related Pain

In patients with PD, previous studies have reported that 20–80% of patients have PD-related pain (Koutoukidis et al., 2021). Although some studies have suggested that STN-DBS can improve PD-related pain, there has been no definite conclusion on the prevalence of PD-related pain after STN-DBS (DiMarzio et al., 2018).

The population morbidity of patients with pain is effectively reduced after DBS. Before DBS, the prevalence of pain was 76% in patients with PD (Figure 6), while after DBS, the prevalence was reduced to 61% (Figure 7). Therefore, we suggest that STN-DBS does not only relieve the pain of patients but can also completely solve the pain problem of some patients. This is of great benefit to improving the quality of life of patients. Although the prevalence of pain increased in an 8 year long-term follow-up study, we suggest that this may be because of the progression of PD (Jung et al., 2015). During 1 and 2 year follow-ups, the prevalence of pain was decreased.

### Limitation

This study has some limitations. As there are few clinical studies on PD-related pain, the current clinical studies on PD-related pain are mostly single-arm ones. Indeed, there are only two high-quality RCTs; therefore, the quality evaluations of the articles included revealed most to be of mid-quality. Nevertheless, this study is the first of its kind to find delayed improvement in PD-related pain, which is important for the programming of DBS. In addition, because there are currently few studies on the treatment of PD-related pain and DBS, we have only included nine studies, which is not enough to analyze the predictive factors of pain. Previous literature reported that duration, depression, age, motor symptoms, and other related factors affect the severity of pain, and we still need to explore this in the future (Fil et al., 2013).

## Conclusions

This study has confirmed the efficacy of STN-DBS on PD-related pain and provides higher-level evidence. Further, this study is the first to find delayed improvement in PD-related pain. We recommend that programming doctors provide patients with advice at the first visit after surgery to reduce the patient's expectation of the acute effect of stimulation. In the later stage of disease progression, patients should be programmed regularly and the parameters should be adjusted to reduce the impact of pain on their quality of life.

## Data Availability Statement

Publicly available datasets were analyzed in this study. This data can be found here: All the datasets can be searched PubMed, Embase, Cochrane Library.

## Author Contributions

All authors listed have made a substantial, direct and intellectual contribution to the work, and approved it for publication.

## Conflict of Interest

The authors declare that the research was conducted in the absence of any commercial or financial relationships that could be construed as a potential conflict of interest.
